# Supplementary material for: Integrative profiling of condensation-prone RNAs during early development
Source: Cell Genom. 2025 Nov 19;6(2):101065. doi: 10.1016/j.xgen.2025.101065 (PMC12903419; doi:10.1016/j.xgen.2025.101065)
Supplement: Document S1. Figures S1–S5 [file mmc1.pdf]

Cell Genomics, Volume *6*

Supplemental information

Integrative profiling of condensation-prone RNAs during early development

Tajda Klobučar, Jona Novljan, Ira A. Iosub, Boštjan Kokot, Iztok Urbančič, D. Marc Jones, Anob M. Chakrabarti, Nicholas M. Luscombe, Jernej Ule, and Miha Modic

Figure S1: Identification of condensation-prone RNAs, related to Figure 1.
(A) Pairwise Pearson correlations between normalised gene-level counts for control, OOPS and semi-extractability assay samples in nPSCs, pPSCs and dPSCs shows high reproducibility among replicates. The dot size is proportional to the Pearson correlation coefficient.
(B) Principal component analysis using normalised gene-level counts using the top 2000 most variable genes separates samples on stage (PC1) and assay (PC2).
(C) smOOPs identification: Venn diagrams showing the overlap between semi-extractability assay and OOPS-enriched genes (differentially expressed vs control; LFC > 1, padj < 0.01) in nPSCs, pPSCs and dPSCs.
(D) Expression of example smOOPs for which fold changes in OOPS vs control are not equally high to the fold changes in semi-extractability assay vs control, and vice-versa. For each stage, the top five genes with highest fold changes in semi-extractability assay data and top five genes with highest fold changes in OOPS data are shown.
(E) lncRNAnet prediction score for TECs (in smOOPs, pooled from all three cell states) with 1 indicating prediction as lncRNA.
(F) Detection of translated ORFs using mESC ribo-seq data[1] for smOOPs lncRNAs and TECs.
(G) Normalised expression profiles (Z-scores of rlog values) across assays in nPSCs for candidate mRNAs analysed by HCR-FISH.
(H) Foci size (area) of all foci detected per transcript, analysed by HCR-FISH in nPSCs with boxplots showing the median and interquartile range.
(I) Quantifications of the HCR-FISH data for smOOPs and non-smOOPs in nPSCs. (Left) Boxplot showing total intensity (mean × area of each foci) normalised to the average intensity of control transcripts for each fluorophore used (*p < 0.05, two-sided Welch’s t-test). (Right) Boxplot showing the fraction of foci for each transcript that are present in the nucleus. n indicates the number of different mRNAs against which the HCR-FISH probes were designed.
(J) Distribution of expression levels in the semi-extractability assay data of transcripts tested using HCR-FISH.
(K) Comparative visualisation of transcriptome data for an example smOOPs that was visualised by HCR-FISH, R3hdm2. Normalised crosslinking signal and coverage across assays was plotted using clipplotr[2].

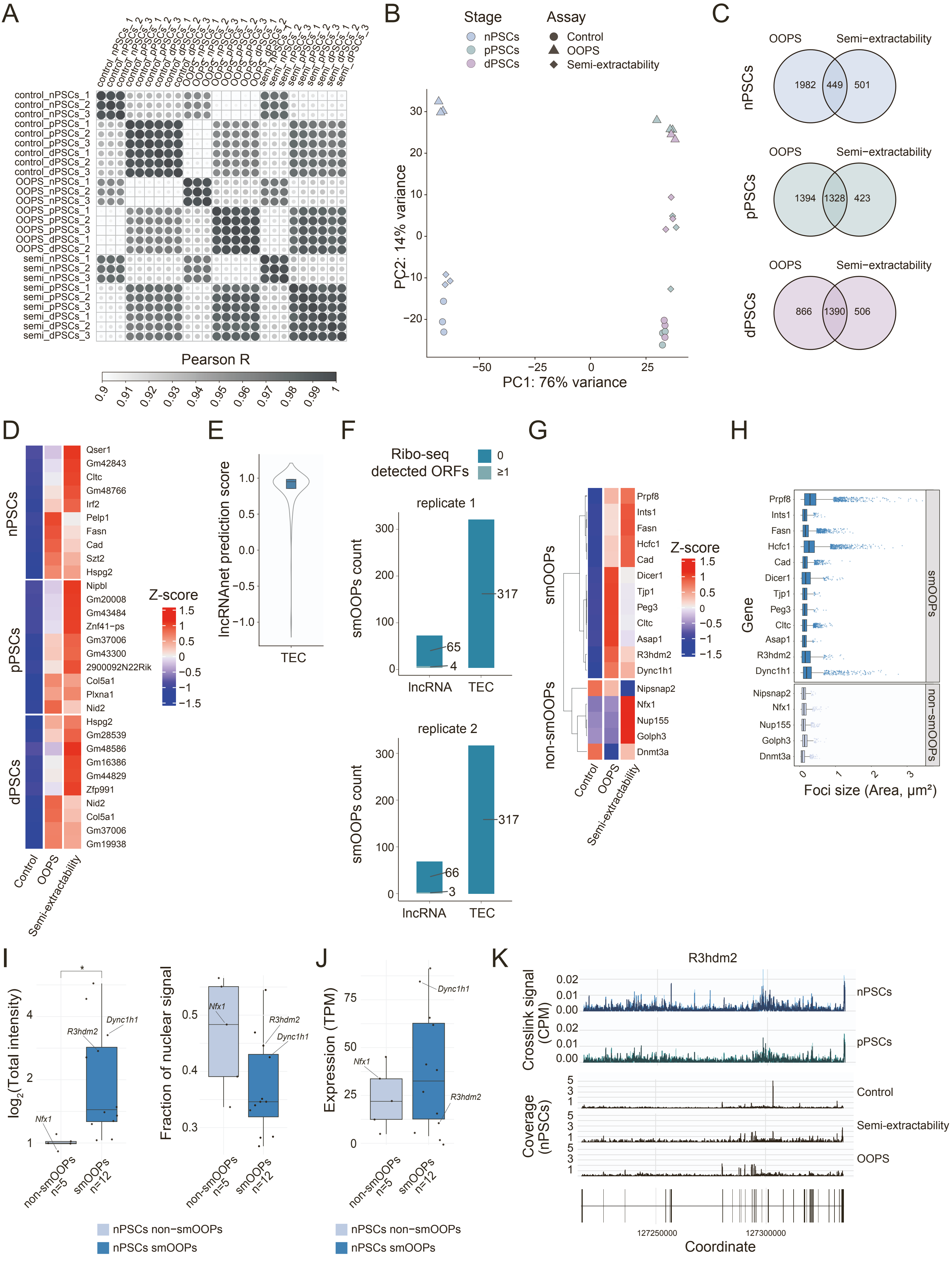

A
Pearson R
B
Stage
nPSCs
pPSCs
dPSCs
Assay
Control
OOPS
Semi−extractability
PC2: 14% variance
PC1: 76% variance
C
OOPS
Semi−extractability
nPSCs
1982
449
501
pPSCs
1394
1328
423
dPSCs
866
1390
506
D
nPSCs
pPSCs
dPSCs
Z-score
Control
OOPS
Semi-extractability
E
lncRNAnet prediction score
TEC
F
Ribo-seq detected ORFs
0
≥1
replicate 1
replicate 2
smOOPs count
lncRNA
TEC
65
4
317
66
3
G
smOOPs
non-smOOPs
Z-score
H
Gene
Foci size (Area, μm²)
smOOPs
non-smOOPs
I
log2(Total intensity)
Fraction of nuclear signal
non-smOOPs n=5
smOOPs n=12
nPSCs non−smOOPs
nPSCs smOOPs
J
Expression (TPM)
K
R3hdm2
Crosslink signal (CPM)
Coverage (nPSCs)
nPSCs
pPSCs
Control
Semi-extractability
OOPS
127250000
127300000
Coordinate

Figure S1

Figure S2: Characterisation of the RIC-seq data and networks in nPSCs and pPSCs, related to Figure 2.
(A) Pearson correlation between samples using normalised intermolecular gene-level hybrid read counts.
(B) PCA of the RIC-seq replicates in nPSCs and pPSCs based on the intermolecular hybrid reads using the top 2000 most variable genes.
(C) Circos plots showing the region links based on intermolecular hybrid reads, with percentages indicating the proportion corresponding to each region type.
(D) Evaluation of the scale-free-like nature of the RIC-seq networks: degree distribution vs gene-counts on a log-log scale. The red line indicates linear fit of the degree distribution on the log-log scale.
(E) Small world properties for the derived RIC-seq networks: Mean global clustering coefficient compared to those of 100 random networks of the same size (equal number of nodes and edges).
(F) Small world properties for the derived RIC-seq networks: Distribution of path lengths in the nPSCs and pPSCs RRI networks, with dashed vertical line indicating the average path length.
(G) Scatter plots showing the positive correlation between degree and expression across gene length quartiles (1-4). Blue and green Pearson coefficients (R) represent nPSCs and pPSCs smOOPs, respectively, while dark grey represents all genes.
(H) Quantile matching to select non-smOOPs with expression levels and gene lengths similar to smOOPs. The 2D density plots illustrate the distribution of all non-smOOPs, smOOPs, and expression- and length-matched non-smOOPs.
(I) Comparison of degree (number of distinct connections with other genes) distribution in the nPSCs and pPSCs RIC-seq networks between smOOPs, all non-smOOPs and the expression- and length-matched non-smOOPs, as identified in panel H.

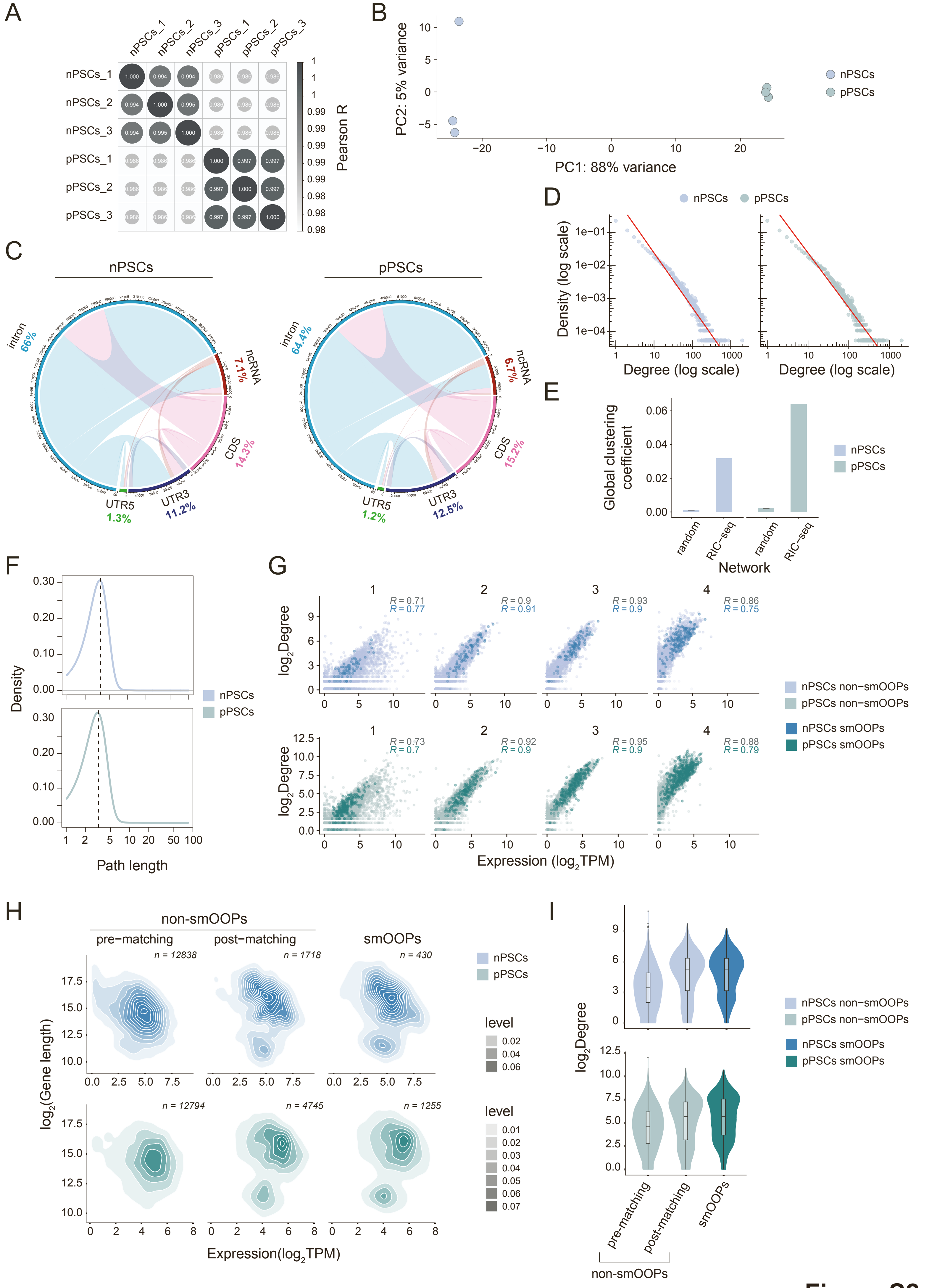

A
nPSCs_1
nPSCs_2
nPSCs_3
pPSCs_1
pPSCs_2
pPSCs_3
Pearson R
B
PC1: 88% variance
PC2: 5% variance
nPSCs
pPSCs
C
nPSCs
pPSCs
intron 66%
ncRNA 7.1%
CDS 14.3%
UTR3 11.2%
UTR5 1.3%
intron 64.4%
ncRNA 6.7%
CDS 15.2%
UTR3 12.5%
UTR5 1.2%
D
Density (log scale)
Degree (log scale)
E
Global clustering coefficient
random
RIC−seq
Network
F
Density
Path length
G
log2Degree
Expression (log2TPM)
nPSCs non−smOOPs
pPSCs non−smOOPs
nPSCs smOOPs
pPSCs smOOPs
H
non-smOOPs
pre−matching
post−matching
smOOPs
n = 12838
n = 1718
n = 430
n = 12794
n = 4745
n = 1255
log2(Gene length)
Expression(log2TPM)
level
I
log2Degree
pre-matching
post-matching
smOOPs
non-smOOPs

Figure S2

Figure S3: Training deep learning models to classify nPSCs smOOPs transcripts, related to Figure 3.

(A) Normalised expression levels for smOOPs and controls in the nPSCs semi-extractability assay data (ns: not significant, $p > 0.05$, two-sided Welch's t-test).

(B) Distribution of full transcript lengths and transcript region-specific lengths for nPSCs smOOPs and controls (**$p < 0.01$; ***$p < 0.001$, two-sided Welch's t-test).

(C) Training and validation loss, accuracy, and AUROC curves over training epochs for models trained on sequence (left) and POSTAR3[3] peaks (right) features. The dashed lines indicate the early stopping points based on validation AUROC performance.

(D) Classification performance of models trained on each individual feature set. Confusion matrices displaying the distribution of true and predicted labels for nPSCs smOOPs and control transcripts.

(E) Venn diagram showing the overlap between nPSCs smOOPs, recovered in the original dataset (first batch) and the second batch.

(F) AUROC curves showing performance on second-batch smOOP classification. The curves illustrate the true positive rate versus false positive rate for three models trained on first-batch data: the baseline length-only model (AUROC = 0.667), the sequence-only model (AUROC = 0.750), and the all-features model (AUROC = 0.785).

(G) AUROC curves showing the effect of masking specific transcript regions (5'UTR, CDS, 3'UTR) on the predictions of models trained solely on either sequence, global iCLIP, POSTAR3[3], and PARIS-Intra[4] features for cluster 1 smOOPs and control transcripts.

(H) Importance scores across transcript positions for Dnmt1 (left) and Tmem265 (right). Importance scores are derived from models trained on either global iCLIP, PARIS-Intra[4] interactions, and the POSTAR3[3] peak dataset, shown in the respective tracks, with POSTAR3[3] RBP importance profiles shown for individual RBPs.

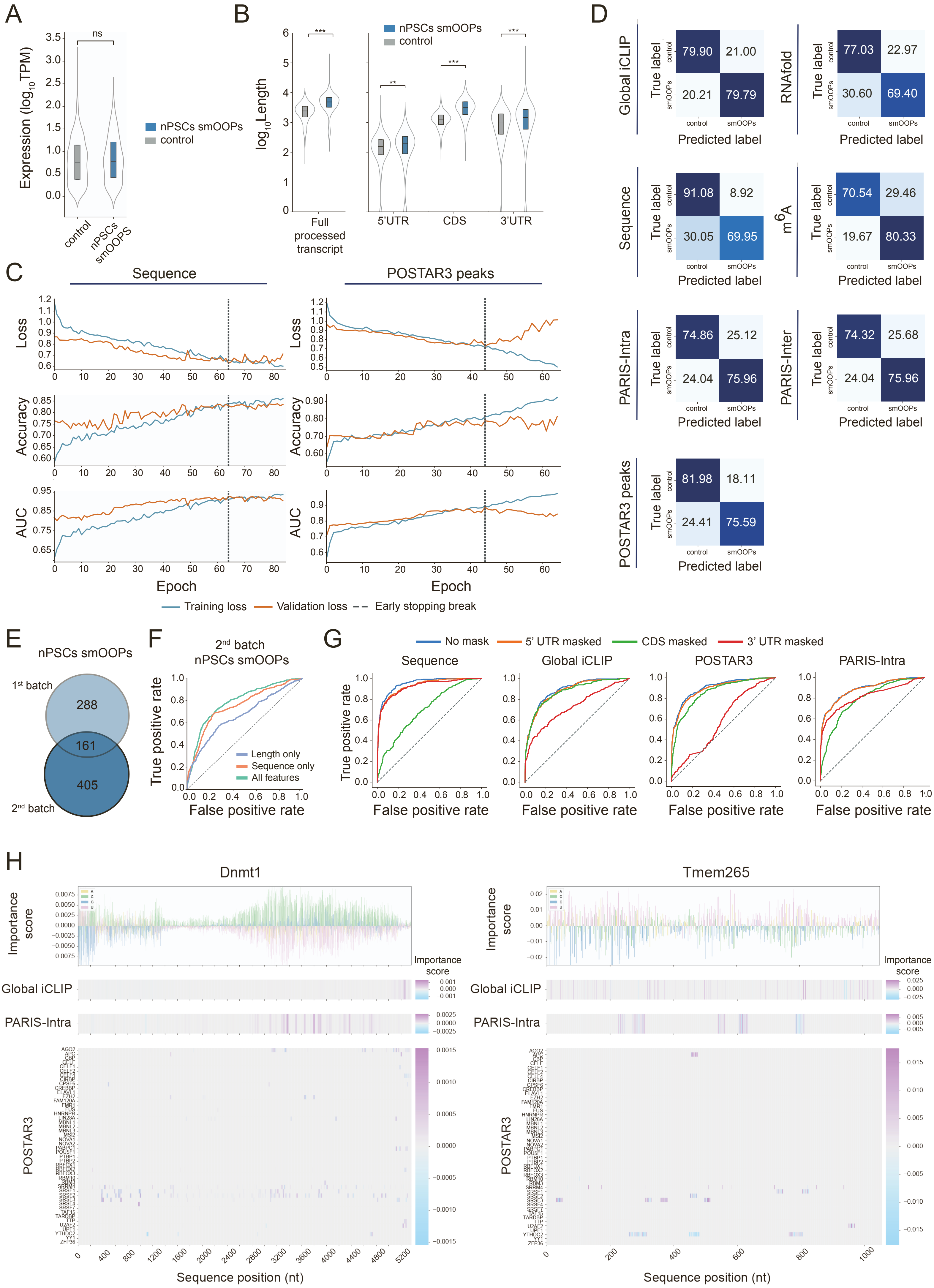
A
ns
Expression (log_{10}TPM)
control
nPSCs smOOPS
nPSCs smOOPs
control
B
***
**
***
***
log_{10}Length
Full processed transcript
5'UTR
CDS
3'UTR
nPSCs smOOPs
control
C
Sequence
POSTAR3 peaks
Loss
Accuracy
AUC
Epoch
Training loss
Validation loss
Early stopping break
D
Global iCLIP
79.90
21.00
20.21
79.79
RNAfold
77.03
22.97
30.60
69.40
Sequence
91.08
8.92
30.05
69.95
m6A
70.54
29.46
19.67
80.33
PARIS-Intra
74.86
25.12
24.04
75.96
PARIS-Inter
74.32
25.68
24.04
75.96
POSTAR3 peaks
81.98
18.11
24.41
75.59
True label
Predicted label
control
smOOPs
E
nPSCs smOOPs
1st batch
288
161
405
2nd batch
F
2nd batch nPSCs smOOPs
True positive rate
False positive rate
Length only
Sequence only
All features
G
No mask
5' UTR masked
CDS masked
3' UTR masked
Sequence
Global iCLIP
POSTAR3
PARIS-Intra
True positive rate
False positive rate
H
Dnmt1
Tmem265
Importance score
Global iCLIP
PARIS-Intra
POSTAR3
Sequence position (nt)

Figure S3

Figure S4: Identifying the positional importance of the most predictive features, related to Figure 4.

(A) Heatmap showing the average individual feature importance scores across all transcripts in cluster 1, divided into 100 bins over the entire transcript, with expanded individual POSTAR3 RBP importance.

(B) As in (A), but for cluster 2.

(C) Heatmap showing the importance scores of the sequence model averaged over a sliding window for individual triplets, binned into 100 bins over the entire transcript and averaged per bin for cluster 1.

(D) As in (C), but for cluster 2.

(E) The difference in average triplet content between cluster 1 nPSCs smOOPs and non-smOOPs for each transcript region.

(F) The difference in average triplet content between cluster 2 nPSCs smOOPs non-smOOPs.

(G) Median global iCLIP signal, normalised for expression and binned (100 bins across the transcripts; pooled from three replicates) for cluster 2 mRNA smOOPs (n=10) and control transcripts. Shaded areas represent 95% confidence intervals per bin, estimated via bootstrapping.

(H) Translation efficiency in nPSCs for smOOPs and non-smOOPs mRNAs obtained by Ribo-seq[5] (ns: not significant, $p > 0.05$, two-sided Welch's t-test).

(I) Half-lives (h) of nPSCs smOOPs mRNAs compared to non-smOOPs, obtained by SLAM-seq[6] (***$p<0.001$; ns: not significant, $p > 0.05$, two-sided Welch's t-test).

(J) Rate constants of decay (k in CPM/h) of nPSCs smOOPs mRNAs compared to non-smOOPs transcripts, obtained by SLAM-seq[6] (**$p < 0.01$; ns: not significant, $p > 0.05$, two-sided Welch's t-test).

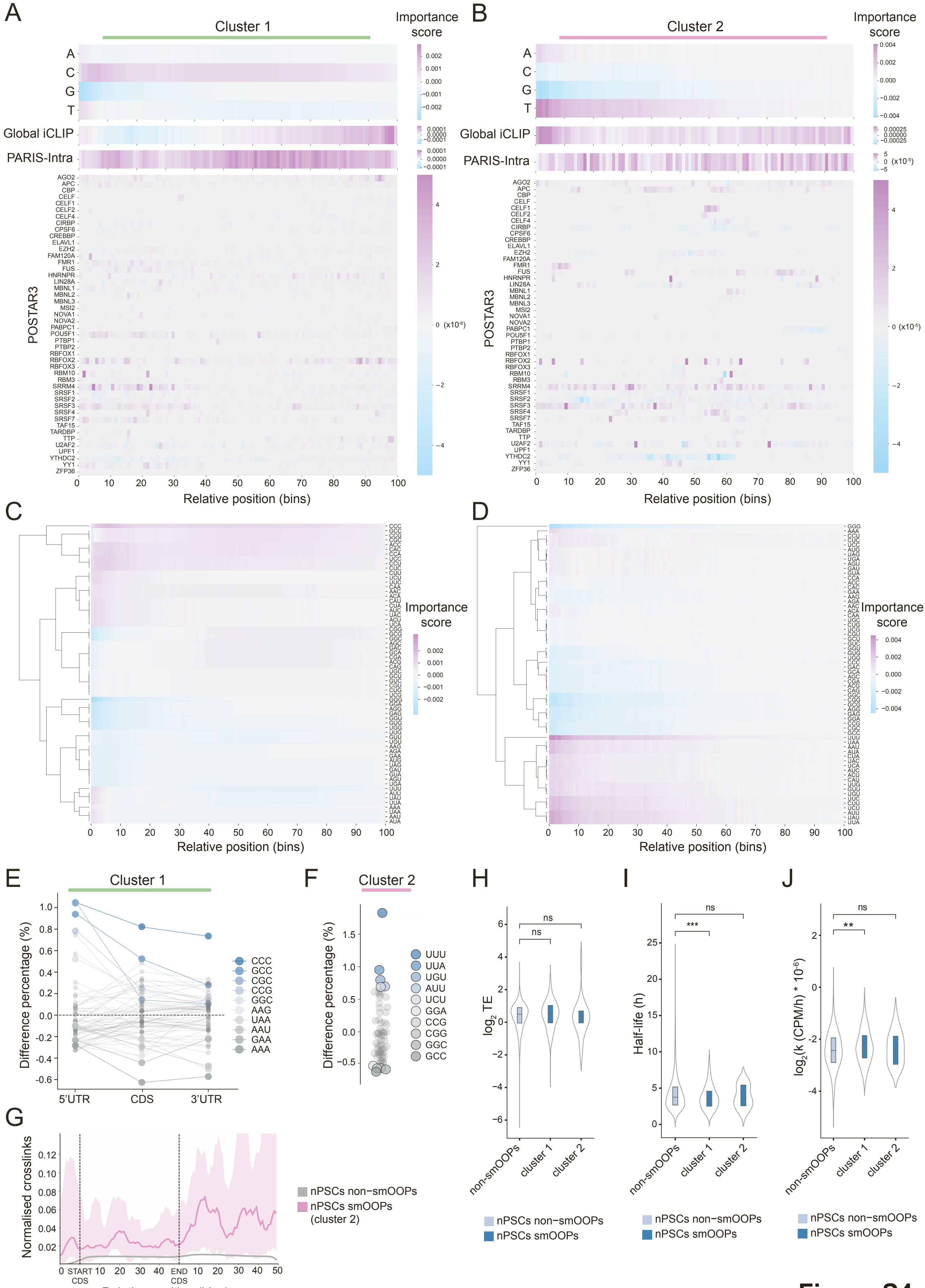

A
Cluster 1
Importance score
Global iCLIP
PARIS-Intra
POSTAR3
Relative position (bins)
B
Cluster 2
C
D
E
Difference percentage (%)
5'UTR
CDS
3'UTR
F
G
Normalised crosslinks
nPSCs non-smOOPs
nPSCs smOOPs (cluster 2)
H
log_2 TE
I
Half-life (h)
J
log_2(k (CPM/h) * 10^{-6})
non-smOOPs
cluster 1
cluster 2
nPSCs smOOPs

Figure S4

Figure S5: Functional and structural characteristics of smOOPs clusters, related to Figure 5.
(A) Scatter plot of UMAP components for all stages smOOPs transcripts based on binned importance scores for sequence only. Each dot represents a transcript, colour-coded by cluster.
(B) Barplot showing the overlap between clusters 1 and 2 of nPSCs smOOPs with the two clusters (C-rich, A/U-rich) obtained by clustering smOOPs from all developmental stages (nPSCs, pPSSc, dPSCs).
(C) Number of smOOPs transcripts in C-rich and A/U-rich clusters across developmental stages.
(D) Gene type composition of C-rich and A/U-rich smOOPs clusters across developmental stages.
(E) Percentage of proteins with IDRs and measures of disorder for nPSCs smOOPs and non-smOOPs, identified from a second batch. Barchart shows the percentage of proteins with IDRs and the box plots show the percentage of disorder in proteins, their mass and PICNIC score (Proteins Involved in CoNdensates In Cells)[7] (***$p < 0.001$; two-sided Wilcoxon rank-sum test for disorder percentage, Welch's two-sided t-test for mass and PICNIC scores).
(F) Relationship between protein disorder and PICNIC scores for proteins encoded by smOOPs in the C-rich and A/U-rich clusters.
(G) Distinct and overlapping GO term enrichments in C-rich and A/U-rich smOOPs clusters.

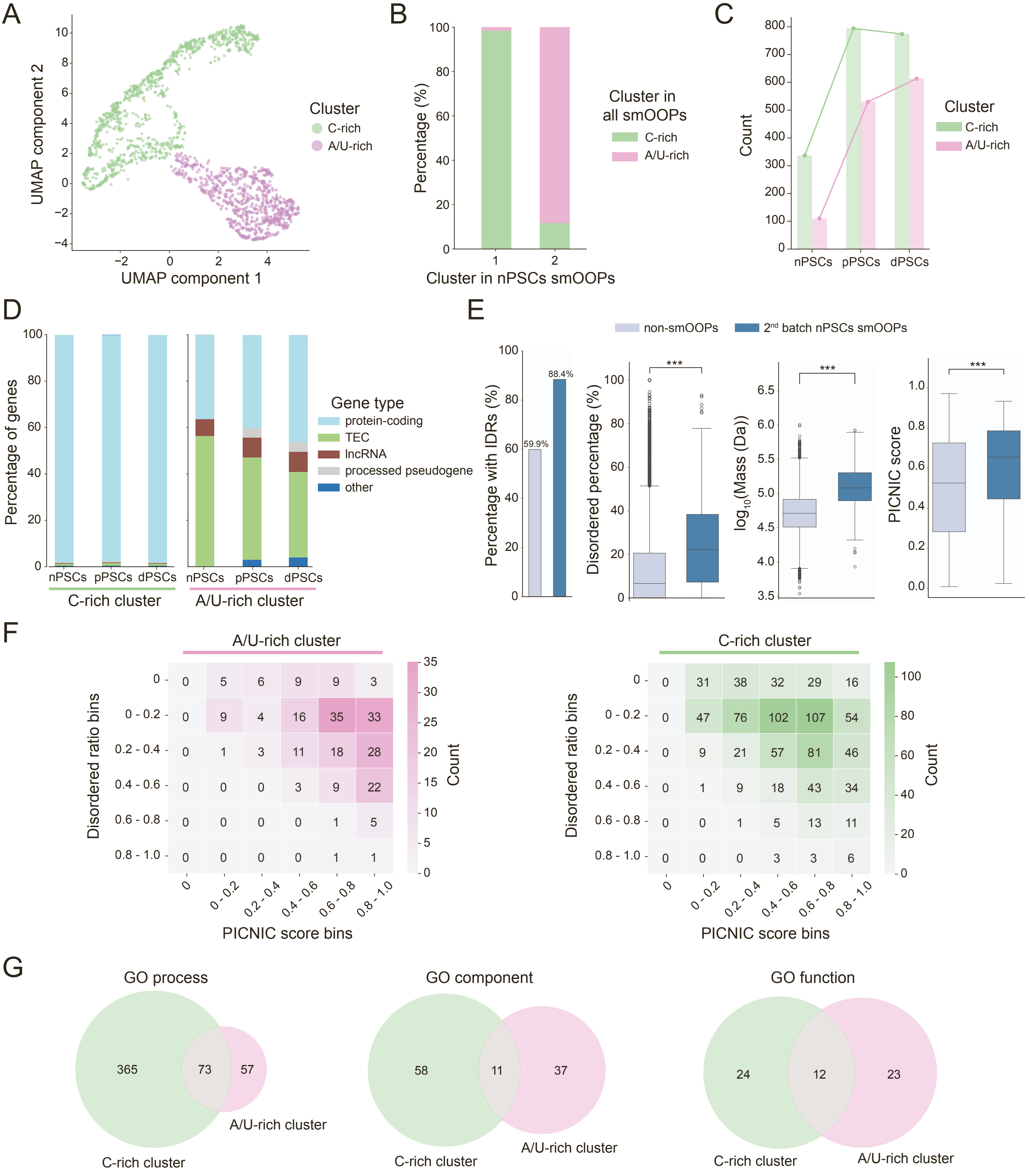

A
UMAP component 2
UMAP component 1
Cluster
C-rich
A/U-rich
B
Percentage (%)
Cluster in nPSCs smOOPs
Cluster in all smOOPs
C-rich
A/U-rich
C
Count
nPSCs
pPSCs
dPSCs
Cluster
C-rich
A/U-rich
D
Percentage of genes
Gene type
protein-coding
TEC
lncRNA
processed pseudogene
other
nPSCs
pPSCs
dPSCs
C-rich cluster
A/U-rich cluster
E
non-smOOPs
2nd batch nPSCs smOOPs
Percentage with IDRs (%)
59.9%
88.4%
Disordered percentage (%)
log10(Mass (Da))
PICNIC score
***
F
A/U-rich cluster
C-rich cluster
Disordered ratio bins
PICNIC score bins
Count
G
GO process
365
73
57
GO component
58
11
37
GO function
24
12
23
C-rich cluster
A/U-rich cluster

Figure S5

Supplemental references.

1. Tuck, A.C., Rankova, A., Arpat, A.B., Liechti, L.A., Hess, D., Iesmantavicius, V., Castelo-Szekely, V., Gatfield, D., and Bühler, M. (2020). Mammalian RNA decay pathways are highly specialized and widely linked to translation. Mol. Cell *77*, 1222–1236.e13.

2. Chakrabarti, A.M., Capitanchik, C., Ule, J., and Luscombe, N.M. (2023). clipplotr- a comparative visualization and analysis tool for CLIP data. RNA *29*, 715–723.

3. Zhao, W., Zhang, S., Zhu, Y., Xi, X., Bao, P., Ma, Z., Kapral, T.H., Chen, S., Zagrovic, B., Yang, Y.T., et al. (2022). POSTAR3: an updated platform for exploring post-transcriptional regulation coordinated by RNA-binding proteins. Nucleic Acids Res. *50*, D287–D294.

4. Lu, Z., Zhang, Q.C., Lee, B., Flynn, R.A., Smith, M.A., Robinson, J.T., Davidovich, C., Gooding, A.R., Goodrich, K.J., Mattick, J.S., et al. (2016). RNA duplex map in living cells reveals higher-order transcriptome structure. Cell *165*, 1267–1279.

5. Ingolia, N.T., Lareau, L.F., and Weissman, J.S. (2011). Ribosome profiling of mouse embryonic stem cells reveals the complexity and dynamics of mammalian proteomes. Cell *147*, 789–802.

6. Herzog, V.A., Reichholf, B., Neumann, T., Rescheneder, P., Bhat, P., Burkard, T.R., Wlotzka, W., von Haeseler, A., Zuber, J., and Ameres, S.L. (2017). Thiol-linked alkylation of RNA to assess expression dynamics. Nat. Methods *14*, 1198–1204.

7. Hadarovich, A., Singh, H.R., Ghosh, S., Scheremetjew, M., Rostam, N., Hyman, A.A., and Toth-Petroczy, A. (2024). PICNIC accurately predicts condensate-forming proteins regardless of their structural disorder across organisms. Nat. Commun. *15*, 10668.
